# Complete genome sequence of plant growth-promoting *Bacillus stratosphericus* AIMST-CREST02 isolated from bulk soil of a paddy field

**DOI:** 10.1128/mra.01137-23

**Published:** 2024-03-20

**Authors:** Sumitra Sivaprakasam, Nur Arisa Mohd Azim Khan, Tan Yee Fan, Yukgehnaish Kumarasan, Thomas Sicheritz-Pontén, Bent Petersen, Erneeza Mohd Hata, Ganesan Vadamalai, Sivachandran Parimannan, Heera Rajandas

**Affiliations:** 1 Centre of Excellence for Omics-Driven Computational Biodiscovery (COMBio), AIMST University, Bedong, Kedah, Malaysia; 2 Department of Plant Protection, Faculty of Agriculture, Universiti Putra Malaysia, Serdang, Selangor, Malaysia; 3 Center for Evolutionary Hologenomics, Globe Institute, University of Copenhagen, Copenhagen, Denmark; 4 Laboratory of Sustainable Agronomy and Crop Protection, Institute of Plantation Studies, Universiti Putra Malaysia, Serdang, Selangor, Malaysia; University of Strathclyde, Glasgow, United Kingdom

**Keywords:** *Bacillus stratosphericus*, plant growth-promoting

## Abstract

Here, we present the complete genome of a plant growth-promoting strain, *Bacillus stratosphericus* AIMST-CREST02 isolated from the bulk soil of a high-yielding paddy plot. The genome is 3,840,451 bp in size with a GC content of 41.25%. Annotation predicted the presence of 3,907 coding sequences, including genes involved in auxin biosynthesis regulation and gamma-aminobutyric acid (GABA) metabolism.

## ANNOUNCEMENT

The members of the genus *Bacillus* are predominantly known for their plant growth-promoting (PGP) traits and can synthesize various beneficial metabolites even under harsh environmental conditions. This makes them an ideal candidate for biofertilizer development (1, 2). Here, we report the complete genome of a potential PGP *Bacillus stratosphericus* AIMST-CREST02 isolated from the bulk soil sample from a high-yielding paddy plot at Kampung Gajah, Perak, Malaysia (4.1841° N, 100.9389° E) (3–5). We collected 30 grams of submerged bulk soil sample at a depth of 0–20 cm on 1 July 2022.

The strain was initially isolated on a nutrient agar plate incubated at 37°C for 24 hours following the serial dilution of 2.5 grams of paddy bulk soil with sterile distilled water. Upon incubation, a single colony was inoculated and cultivated in 5 mL of fresh Luria-Bertani broth for 24 hours at 37°C. The genomic DNA was then extracted using the GeneJET Genomic DNA Purification Kit (ThermoFisher) following the manufacturer’s instructions.

A non-fragmented and non-size-selected DNA library was prepared with SQK-NBD114.24 native barcoding kit. Sequencing was performed with Oxford Nanopore Technology’s MinION platform, using an R10.4.1 flow cell. Guppy v6.5.7 (high-accuracy model), a recurrent neural network-based basecaller, was used (6). A total of 277,493 raw reads were generated with a read length *N*_50_ value of 7,197. The raw reads were trimmed with Porechop v0.2.4 (7) and *de novo* assembled using Flye v2.9.2 (8). The resulting unrotated contig was subsequently polished using Medaka v1.8.1 (9). The publicly available, linked, resource (CP137077) was then annotated with PGAP v6.6 (10). Default parameters were used for all the analyses unless otherwise stated.

Visualization of the assembly graph with Bandage v0.9.0 (11) indicated that the resulting contig was a single circular chromosome (in agreement with Flye’s output) of 3,840,451 bp with a GC content of 41.25%, *N*_50_ length of 3,840,451, and 448× mean coverage. PGAP predicted that the genome encodes 4,017 genes including 3,907 coding sequences, 24 rRNA genes (5S, 16S, and 23S), 81 tRNAs, 5 non-coding RNAs, and 314 pseudogenes. The similar genome finder service on BV-BRC v3.33.16 (12) identified *Bacillus stratosphericus* LAMA 585 (APAS00000000) to be the closest strain to the assembled genome, and an average nucleotide identity analysis on EzBioCloud (update 2023.08.23) (13) indicated a high sequence similarity of 98.39% between the genomes.

In addition, the presence of several PGP-attributable genes was predicted using the RAST toolkit (RASTtk) (14). The complete genome possessed genes that regulate auxin biosynthesis (anthranilate phosphoribosyltransferase, phosphoribosyl anthranilate isomerase, and tryptophan synthase) and GABA metabolism (gabR). The presence of both GABA and auxin-related genes implies that the isolate will be efficient in alleviating plant stress while enhancing plant growth and development (15). The genome also possessed genes associated with siderophores’ production, especially bacillibactin, known to improve iron availability (16, 17). Genes associated with cobalt-zinc-cadmium resistance and volatile organic compound (VOC) metabolism (acetoin and butanediol) beneficial to plants were also found. Therefore, this strain can be used as a potential candidate bacterium for biofertilizer development.

## Data Availability

The complete genome sequence of *Bacillus stratosphericus* AIMST-CREST02 has been deposited in NCBI GenBank under the BioProject ID PRJNA1028329, BioSample ID SAMN37821796, SRA ID SRR26661738, and GenBank accession CP137077. The annotation output of RASTtk has been deposited under 10.6084/m9.figshare.25233520.
